# A Pilot Pre and Post 4 Week Intervention Evaluating the Effect of a Proprietary, Powdered, Plant Based Food on Micronutrient Status, Dietary Intake, and Markers of Health in a Healthy Adult Population

**DOI:** 10.3389/fnut.2022.945622

**Published:** 2022-07-11

**Authors:** Matthew D. Wilcox, Peter I. Chater, Kyle J. Stanforth, Rebecca Williams, Iain A. Brownlee, Jeffrey P. Pearson

**Affiliations:** ^1^Biosciences Institute, Medical School, Newcastle University, Newcastle upon Tyne, United Kingdom; ^2^Aelius Biotech Ltd., The Biosphere, Newcastle upon Tyne, United Kingdom; ^3^Huel Ltd., Tring, United Kingdom; ^4^Department of Applied Sciences, Northumbria University, Newcastle upon Tyne, United Kingdom

**Keywords:** micronutrient, Huel, vitamin status, micronutrient status, intervention study

## Abstract

**Background:**

A “balanced, adequate, and varied diet” is recommended as the basis of nutritionally sound diet by the World Health Organisation and national public health agencies. Huel is a proprietary, on-the-go, powdered, plant based food, providing all 26 essential vitamins and minerals, protein, essential fats, carbohydrate, fibre, and phytonutrients.

**Objectives:**

Assessing the effect of solely consuming Huel on micronutrient status, dietary intake and markers of health was achieved through a 4-week intervention of solely Huel powder.

**Methods:**

Habitual energy intake was assessed through a one-week lead in period with healthy adult participants (aged 18 or over) logging their food intake, after which only Huel was consumed for 4 weeks. Blood samples and body composition was assessed before and after the lead in week as well the end of the intervention. Thirty participants were recruited with 20 (11 females, median age 31, range 22–44) completing the study, 19 sets of blood samples were collected. 22 blood markers were analysed along with weight, BMI, waist circumference, visceral adipose tissue (VAT), and body composition. All blood micronutrients, except for Thyroid Stimulating Hormone and choline were sent to Royal Victoria Infirmary NHS, Newcastle Laboratory (Newcastle upon Tyne, United Kingdom) for analysis.

**Results:**

Fourteen of the parameters significantly changed over the course of the study with circulating haemoglobin, iron, vitamins B12 and D as well as selenium significantly increasing (*p* < 0.05). HbA1c, total and non-HDL cholesterol, vitamins A and E, potassium, BMI, VAT, and waist circumference all significantly decreased (*p* < 0.05) post intervention.

**Conclusion:**

Although energy intake decreased during the intervention period, the adherence to recommended micronutrient intake, as quantified by the dietary Total Adherence Score, significantly increased which tallies with the preservation or improvement of micronutrient status. This study potentially demonstrates that consuming only Huel for 4 weeks does not negatively affect micronutrient status.

## Introduction

The World Health Organisation and public health agencies recommend a “balanced, adequate, and varied diet” for good health and wellbeing (1, 2). A diverse and varied diet provides the full range of nutrients and macronutrients required for optimal health, and it is well understood that both nutritional insufficiencies and excesses can be detrimental to health (3). Both food- and nutrient-based dietary guidelines are based on key concepts of providing sufficient energy and nutrients to meet the requirements of almost everyone within a given population (4). Over nutrition (e.g., excess body weight) along with undernutrition (e.g., iron deficiency) are significant challenges to global health (5, 6), with poor diet now the major cause of non-communicable disease globally (7). Improving health through dietary modification is thought to be an achievable way in which to reduce healthcare costs globally while increasing quality of life, functionality, and productivity for its citizens.

Meal replacements have been developed over many years, with the key components being described by Stephens et al. in 1969 (8) but their concept was much earlier with their evolution rapidly progressing during the late 1950s “Space Race” (9). Meal replacement products have previously been defined as “a commercially available product, fortified with minerals and vitamins designed to replace one or two meals per day with at least one meal consumed as normal food, for example with meat and vegetables and consumed as part of an energy restricted diet” (10). However, Huel is not sold specifically as a meal replacement, or a supplement, but it is possible consumers may use it as such.

Huel’s longest standing product is Huel Powder is a commercially available food that has been formulated to provide carbohydrate, protein, fat, and fibre and all essential vitamins and minerals at levels at least sufficient to meet recommended intake for an equivalent meal (11). This is achieved through a diverse ingredient list that provides protein (pea protein, brown rice protein, and oats), carbohydrates (from oats and tapioca), fats (from flaxseed, sunflower seeds, and coconut), fibre (from oats and flaxseed), and supplemented with certain micronutrients and phytonutrients not present in sufficient amounts in other ingredients. With each 100 g serving of Huel containing 400 calories, five servings per day would be required to achieve 2,000 calories per day. Huel is intended to replace food and does not strictly fit the full definition of ‘meal replacement’ as it is not part of an energy restricted diet. While the product was formulated to be nutritionally complete, there is a need for further understanding of how such a product may impact parameters of health and dietary habits within free-living adults. To the authors’ knowledge, there has been no previous study evaluating the impact of Huel products on nutritional status, health outcomes and dietary intake.

The aim of this study was to investigate the effects of a diet consisting solely of Huel powdered product on nutritional status and anthropomorphic measures of health [weight, waist circumference, BMI, and visceral adipose tissue (VAT)].

The hypothesis of this study is that a diet of 100% Huel maintains the levels of circulating micronutrients at desirable levels.

## Materials and Methods

### Materials

Huel Powder v3.0 was supplied by Huel Ltd. (Tring, United Kingdom) in five flavours; vanilla, chocolate, mint choc, berry, and banana. Vacutainer^®^ Blood Collection Tubes, holders, and butterfly needles were purchased from BD (Franklin Lakes, NJ, United States). Seca mBCA 515 Body Composition Analyzer (Hamburg, Germany) was used for, weight, BMI, and body composition of participants. The study involved recruitment of 30 generally healthy male and (non-pregnant, non-lactating) female participants aged 18 or over. Potential volunteers were asked if they were taking medication for long term illnesses such as high blood pressure, heart disease, or medication that affects liver or kidney function, or had any known food allergies or dietary supplements. Participants were provided an honorarium for their involvement in the study.

### Methods

**FIGURE 1:**
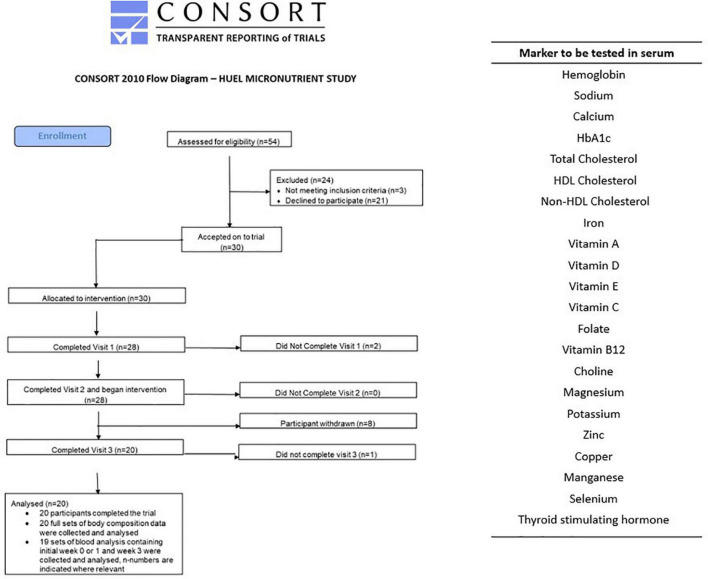
CONSORT diagram and list of blood biomarkers assessed at each of the three visits. The two participants who withdrew at the second visit were due to failed phlebotomy. Of the eight who withdrew before the third visit two gave no reason, two had difficulty maintaining diet, one due to mood changes, one due to stomach issues, and two due to headaches.

The study was an open label trial, with one intervention arm. Participants had a lead-in week to the study to document their normal dietary habit. Following baseline blood sampling participants were then asked to consume only the Huel Powder provided to them by the study team for 4 weeks, with only water, black coffee, and non-citrus teas being the exceptions. All food during the 5 week study was recorded on a food diary and adherence was verified through review of online food records (see below for further details) which the research team regularly reviewed to ensure compliance. Food diary data from the initial week was used to calculate the recommended amount of Huel volunteers should consume over the intervention period. Fasted blood samples (12 h overnight) were taken at the start of study, (week 0), end of lead-in week (week 1), and end of the intervention arm (week 5). Samples were taken after an overnight fast, as is consistent with the literature. The circulating markers listed in Figure 1 were quantified.

The BMI of participants was calculated at each of the three study visits along with the VAT and body composition. Waist circumference and body weight were also measured. All anthropomorphic measurements were taken whilst volunteers were in light clothing but with the shoes and socks removed. Waist circumference was measured over thin clothes between of the bottom ribs and the top of the hips.

The study was run and coordinated from Newcastle University Sport Centre, with 54 potential participants contacted and 30 accepted and enrolled on to the trial. From the 30 participants recruited and enrolled, 20 completed the study (11 female, median age 31, range 22–44) but only 19 sets of blood samples were collected (11 female). This information is represented in the consort diagram in Figure 1. This study was conducted according to the guidelines laid down in the Declaration of Helsinki and all procedures involving human subjects were approved by the Faculty of Medical Sciences Research Ethics Committee, part of Newcastle University’s Research Ethics Committee, study number 2003/4657. Written informed consent was obtained from all subjects. This study was registered on ClinicalTrials.gov, Identifier: NCT04734938 as a simple before and after treatment, 4-weeks intervention with Huel with a food diary completed online throughout the study.

### Blood Biomarker Analysis

Blood samples for thyroid stimulating hormone and choline were collected in sterile vacutainers containing an anticoagulant of K2 Potassium salt of EDTA (Ethylene Diamine Tetra Acetic acid). Samples were centrifuged at 4°C for 15 min at 1,500 *g* and the supernatant collected and stored at –20°C until required.

Samples were analysed for thyroid stimulating hormone content using Human Thyroid Stimulating Hormone ELISA Kit (ab100660) (Abcam, Cambridge, United Kingdom) as per the manufacturers protocol and further assessed for choline content using the Total Choline Assay Kit (ab219944) (Abcam, Cambridge, United Kingdom), as per the manufacturers protocol.

All other samples analysis was performed by Newcastle Laboratories (The Newcastle upon Tyne Hospitals, NHS Foundation Trust, United Kingdom) to standard protocols. All samples were kept on ice and delivered to the Laboratory within 2 h of being taken.

### Dietary Data Analysis

Dietary intake data were originally collected from participants through use of online food diaries (MyFitnessPal, San Fransisco, US). Participants were requested to provide a full week of dietary intake data prior to their baseline visit. Participants were also instructed to maintain food diary records of Huel usage throughout their 4-week intervention period. Data from these food records were processed using Microdiet dietary software v2.8 (Downlee Systems, Salford, United Kingdom), with compositional data from the 2015 Composition of Foods Integrated Datasets (12), with additional information provided through United States, Italian, and South Asian databases where relevant, within manufacturers compositional data added separately for intervention foods (13). Dietary data were converted into a daily mean (±SD) intake to support consideration of adequacy of dietary intake.

Pre-baseline and intervention estimate of daily energy and nutrient intake were compared against age- and sex-specific guidelines for United Kingdom populations. To consider whether the intervention improved overall dietary habit across participants, a scoring system was developed for 25 component dietary factors (see Table 1 for further detail of how these scores were calculated in each instance), with an unweighted total of scores being used to assess overall adherence to energy- and nutrient-based dietary guidelines. These elements are henceforth referred to as “component adherence scores” and “total adherence scores” respectively.

**TABLE 1:** Approach to scoring component nutrient and energy intake and defining component adherence and total dietary adherence scores.

	Maximal score (100) if intake		Minimal score (0) if intake	
	Male	Female	Male	Female
kcal/day	=2,500.0	=2,000.0	≥3,750.0 or ≤1,666.7	≥3,000.0 or ≤1,333.3
Protein (g/day)	≥55.5	≥45.0	≤40.5	≤25.3
Fat (g/day)	≤48.5	≤39.0	≥97.0	≥78.0
CHO (g/day)	≥333.0	≥267.0	≤116.0	≤87.0
Fibre (g/day)	≥30.0	≥30.0	≤7.5	≤7.1
Na (mg/day)	≤1,200.0	≤1200.0	≥2,400.0	≥2,400.0
K (mg/day)*	≥3,500.0	≥3500.0	≤2,000.0	≤2,000.0
Ca (mg/day)*	≥700.0	≥700.0	≤400.0	≤400.0
Mg (mg/day)	≥300.0	≥270.0	≤190.0	≤150.0
P (mg/day)	≥550.0	≥550.0	≤275.0	≤275.0
Fe (mg/day)*	≥8.7	≥14.8	≤4.7	≤8.0
Cu (mg/day)	≥1.2	≥1.2	≤0.6	≤0.6
Zn (mg/day)*	≥9.5	≥7.0	≤5.5	≤4.0
Se (μg/day)*	≥75.0	≥60.0	≤40.0	≤40.0
Cl (mg/day)	≤1,250.0	≤1250.0	≥2400.0	≥2,400.0
I (μg/day)*	≥140.0	≥140.0	≤70.0	≤70.0
Vitamin B1 (mg/day)	≥1.0	≥0.8	≤0.5	≤0.4
Vitamin B2 (mg/day)*	≥1.2	≥1.1	≤0.8	≤0.8
Vitamin B6 (mg/day)	≥1.4	≥1.2	≤0.7	≤0.6
Vitamin B12 (mg/day)	≥1.5	≥1.5	≤0.75	≤0.75
Vitamin C (mg/day)	≥40.0	≥40.0	≤20.0	≤20.0
Vitamin D (μg/day)*	≥10	≥10	≤5.0	≤5.0
Vitamin A (μg/day RetEq)*	≥700.0	≥600.0	≤300.0	≤250.0
Niacin (mg/day)	≥16.5	≥13.2	≤8.3	≤6.6
Folate (μg/day)*	≥200.0	≥200.0	100.0	100.0

*Data for adults aged 19–64 years taken from Public Health England (40). Where Lower Reference Nutrient Intake data are available (https://assets.publishing.service.gov.uk/government/uploads/system/uploads/attachment_data/file/699242/NDNS_yr_7_to_8_statistics.xlsx), these have been used as the minimal score value (denoted by asterisk above). Protein, carbohydrate, and fibre (as estimated by AOAC method) use minimal cut-offs based on 2.5th percentile of intake. Other cut-offs used relate to 50% higher or lower than recommended intake.*

### Statistical Analysis

Data were analysed using GraphPad Prism 8 (San Diego, CA, United States). Data are presented as the mean ± standard deviation, unless otherwise stated. A repeat measures one way ANOVA test with multiple comparison was used to assess statistical changes between timepoints measured for micronutrients in blood samples.

## Results

Blood samples collected from the 19 participants who completed the study and provided sufficient sample were analysed for dietary factors listed in Figure 1. A summary of the changes measured between each of the three timepoints (week 0, 1, and 5) are seen in Table 2. Blood concentrations/biomarkers of nutritional status of eleven of the 21 micronutrients significantly changed (*p* < 0.05) after consumption of the intervention with five increasing and six decreasing significantly.

**TABLE 2:** Statistical changes in micronutrients comparing weeks, 0, 1 and 5.

	Week 0	Week 1	Week 5
Haemoglobin (μmol/L)	141.5 ± 18.20^ab^	139.4 ± 16.72^a^	143.1 ± 13.94*^b^*
Sodium (μmol/L)	140.3 ± 1.1^a^	139.7 ± 1.6^a^	139.7 ± 1.6^a^
Calcium (μmol/L)	2.33 ± 0.06^a^	2.34 ± 0.07^a^	2.34 ± 0.07^a^
HbA1c (mmol/mol)	34.4 ± 4.99^a^	35.0 ± 4.62^a^	32.8 ± 4.01^b^
Total Cholesterol (mmol/L)	4.70 ± 1.19^a^	4.75 ± 1.05^a^	3.68 ± 0.74^b^
HDL cholesterol (mmol/L)	1.55 ± 0.31^a^	1.57 ± 0.36^a^	1.45 ± 0.27^a^
Non-HDL cholesterol (mmol/L)	3.13 ± 1.32^a^	3.18 ± 1.25^a^	2.24 ± 0.78^b^
Iron (μmol/L)	17.89 ± 8.86^ab^	14.47 ± 4.34^a^	18.32 ± 6.19^b^
Vitamin A (μmol/L)	2.03 ± 0.41^a^	2.04 ± 0.40^a^	1.75 ± 0.38^b^
Vitamin D (μmol/L)	46.63 ± 17.65^a^	45.84 ± 18.85^a^	54.00 ± 17.44^b^
Vitamin E (μmol/L)	31.46 ± 10.40^a^	31.69 ± 12.35^a^	22.01 ± 3.83^b^
Vitamin C (μmol/L)	52.27 ± 25.06^a^	59.83 ± 25.19^a^	63.16 ± 27.12^a^
Folate (μmol/L)	9.81 ± 5.78^a^	8.26 ± 3.97^a^	10.34 ± 4.76^a^
Vitamin B12 (pmol/L)	375.9 ± 187.4^a^	352.5 ± 171.1^a^	443.8 ± 122.9^b^
Choline (μmol/L)	1.85 ± 0.69^a^	2.23 ± 0.84^a^	2.41 ± 0.69^a^
Magnesium (mmol/L)	0.84 ± 0.05^a^	0.85 ± 0.06^a^	0.85 ± 0.05^a^
Potassium (mmol/L)	4.40 ± 0.31^a^	4.41 ± 0.37^a^	4.15 ± 0.28 + b
Zinc (μmol/L)	13.11 ± 1.56^a^	12.49 ± 1.61^a^	13.08 ± 1.81 a
Copper (μmol/L)	14.59 ± 2.60^a^	14.37 ± 2.70^a^	14.84 ± 2.95^a^
Manganese nmol/L	187.6 ± 57.43^a^	175.0 ± 33.78^a^	182.7 ± 46.00^a^
Selenium (μmol/L)	1.16 ± 0.27^a^	1.14 ± 0.26^a^	1.40 ± 0.29^b^
TSH (ng/ml)	73.9 ± 60.96^a^	47.40 ± 34.05^a^	41.54 ± 60.15^a^

*Weeks with the same letter are not statistically different, weeks with different letters are significantly different. For example, haemoglobin, week 0 and week 1 are not different, week 0 and week 5 are not different, but week 5 shows a significant increase in levels compared to week 1. TSH, thyroid stimulating hormone.*

Results presented below are of analytes where statistically significant changes were observed, data for micronutrients that did not statistically change over the course of the study are presented in the supplementary section (Appendix A). There were no statistical changes between the measurement taken before the baseline week and after for all the micronutrients that were measured.

**FIGURE 2:**
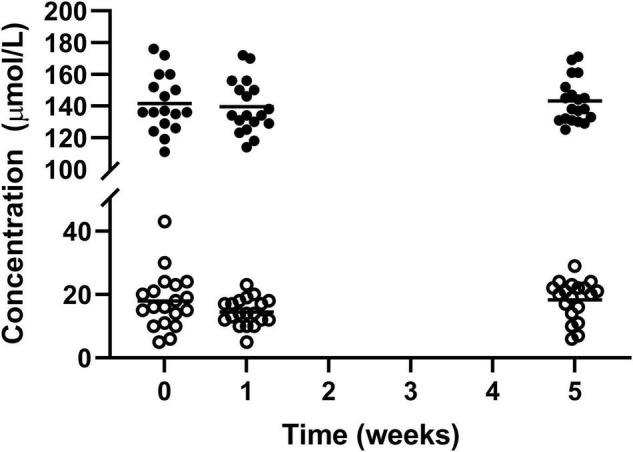
Concentration of circulating haemoglobin l and iron ¢; for the 19 participants who completed the study. The mean of each measurement shown as a solid black line. There were statistical increases for both haemoglobin and iron from week 1 to week 5.

Haemoglobin and Iron both were statistically higher after the intervention with Huel as the sole dietary source of iron compared to week 1 (139.4 ± 16.72 vs 143.1 ± 13.94 and 14.47 ± 4.34 vs 18.32 ± 6.19 μmol/L, respectively) but not when compared to the week 0 measurement (141.5 ± 18.20 1: μmol/L and 17.89 ± 8.86 μmol/L, respectively) (Figure 2).

**FIGURE 3:**
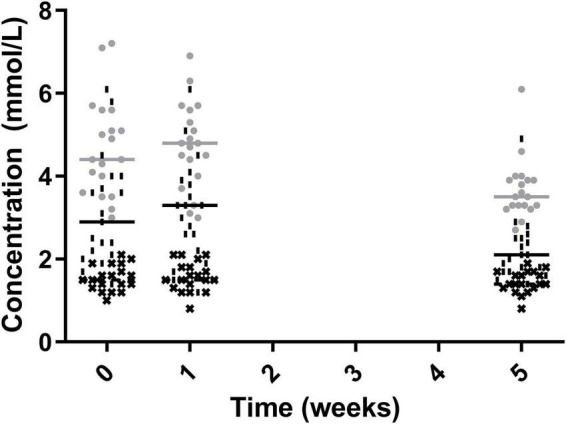
Total Cholesterol (•), Non-HDL Cholesterol (|), HDL cholesterol (✖), for the 19 participants who completed the study. The mean of each measurement shown as a solid grey line for total cholesterol and a solid black line for non-HDL cholesterol and HDL cholesterol.

Total cholesterol and non-HDL cholesterol were significantly lower post-intervention with week 5 measurements being significantly lower than both week 0 and week 1 (4.70 ± 1.19 vs 4.75 ± 1.05 vs 3.68 ± 0.74 for total cholesterol and 3.13 ± 1.32 vs 3.18 ± 1.25 vs 2.24 ± 0.78 for non-HDL cholesterol week 0, 1, and 5, respectively). The level of HDL cholesterol did not significantly change across time points (1.55 ± 0.31 vs 1.57 ± 0.36 vs 1.45 ± 0.27 mmol/L for week 0, 1, and 5, respectively) (Figure 3).

**FIGURE 4:**
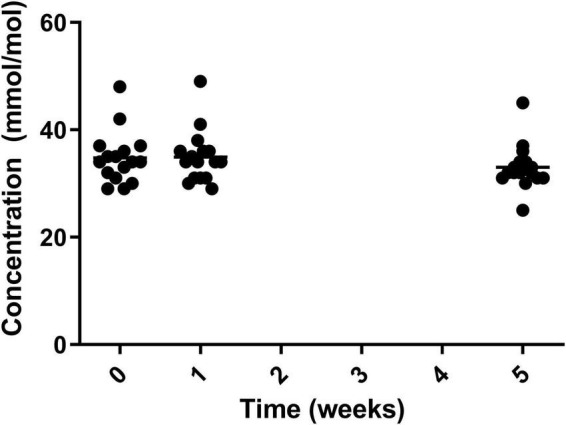
HbA1c for the 19 participants who completed the study. The mean of each measurement shown as a solid black line.

Glycated haemoglobin (HbA1c) Can be used as a marker to assess blood glucose levels over a sustained period (three months) which should ideally be below 42 mmol/mol (6%). The level of HbA1c significantly reduced over the course of the study with significant reduction from both week 0 (34.4 ± 4.99 mmol/mol) and week 1 (35.0 ± 4.62 mmol/mol) to week 5 (32.8 ± 4.01 mmol/mol) (Figure 4).

**FIGURE 5:**
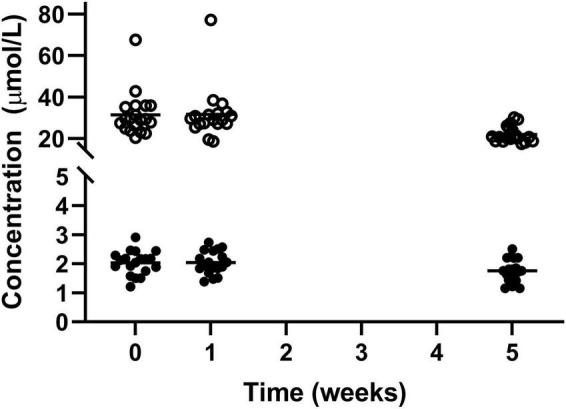
Circulating vitamin A (•) and vitamin E (**○**) for the 19 participants who completed the study. The mean of each measurement shown as a solid black line.

There were significant reductions in blood vitamin A and E status over the course of the study, with vitamin A reducing from its peak of 2.04 ± 0.40 μmol/L in week 1 to 1.75 ± 0.38 μmol/L in week 5. Similarly for vitamin E decreasing from its peak at week 0 (31.46 ± 10.40 μmol/L) to in week 5 (22.01 ± 3.83) μmol/L (Figure 5). However, measurements for both vitamin A and E remained within normal range.

**FIGURE 6:**
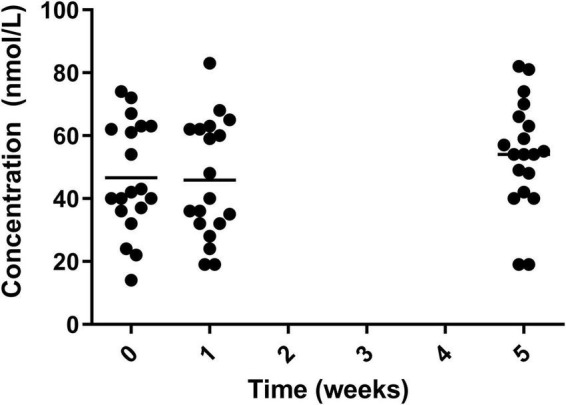
Circulating vitamin D for the 19 participants who completed the study. The mean of each measurement shown as a solid black line.

Levels of vitamin D significantly increased from the two baseline measurements to the final measurement at week 5. The mean level significantly increased from 46.63 ± 17.65 μmol/L in week 0 and 45.84 ± 18.85 μmol/L in week1 to 54.00 ± 17.44 μmol/L in week 5 (Figure 6).

**FIGURE 7:**
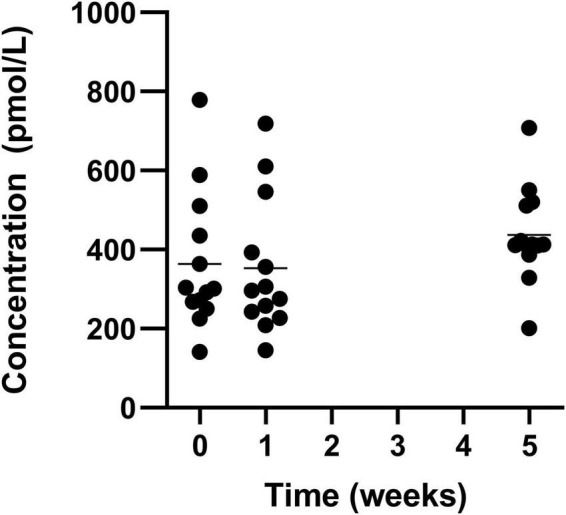
Circulating vitamin B12 for the 19 participants who completed the study. The mean of each measurement shown as a solid black line.

There was a significant increase in mean levels of vitamin B12, with an increase from the lowest level (week 1) of 375.9 ± 187.4 pmol/L to 443.8 ± 122.9 pmol/L in week 5 (Figure 7).

**FIGURE 8:**
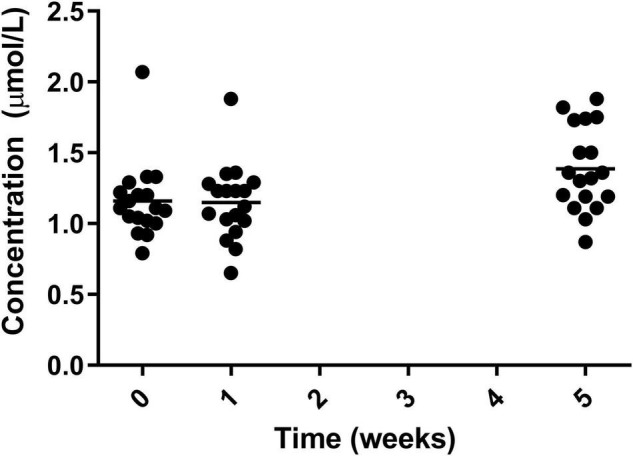
Circulating selenium for the 19 participants who completed the study. The mean of each measurement shown as a solid black line.

A significant increase was also seen in selenium from both week 0 and 1 to week 5 with the greatest increase coming from week 1 (1.14 ± 0.26 μmol/L) to week 5 (1.40 ± 0.29 μmol/L) (Figure 8).

**FIGURE 9:**
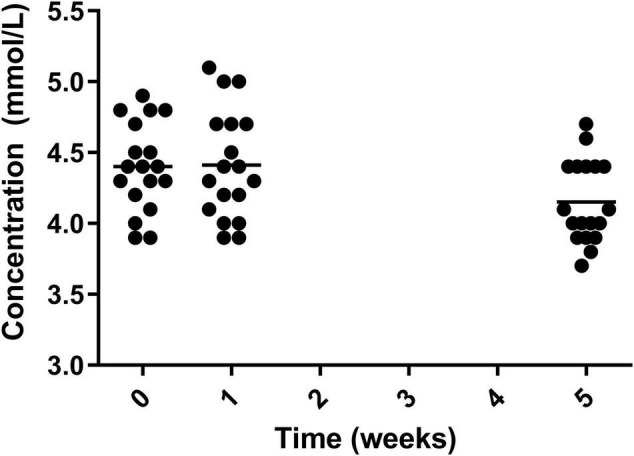
Circulating potassium for the 19 participants who completed the study. The mean of each measurement shown as a solid black line.

Levels of potassium significantly decreased from both week 0 and week 1 to week 5, decreasing from 4.40 ± 0.31 mmol/L in week 0 and 4.41 ± 0.37 mmol/L in week 1 to 4.15 ± 0.28 mmol/L in week 5 (Figure 9). The measurement at week 5 remained within normal range.

**FIGURE 10:**
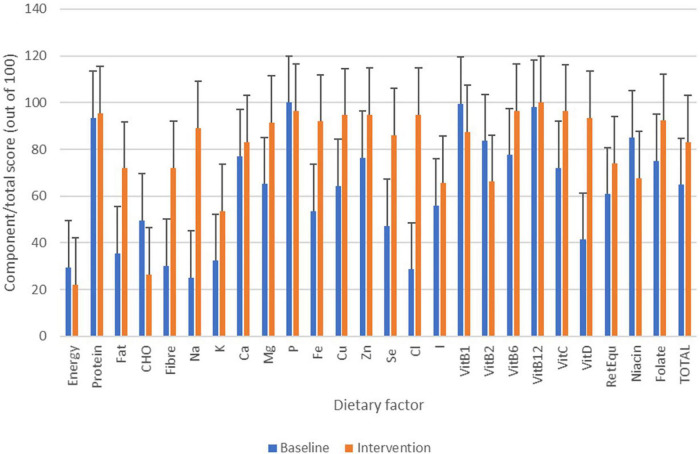
Changes in micronutrient/energy intake based on food diary data over the course of the study. Bars in blue show micronutrient/energy intake from the baseline week with bars in orange showing micronutrient/energy intake from Huel diet only.

From food diary data, micronutrient and energy intake was monitored and normalised for recommended intake across ages and sex (Figure 10) (4) (the initial week vs 4 weeks of intervention). During the intervention, no one met their population level recommendation for energy intake with low intake of Huel servings. Despite low energy intake, intake of most nutrients improved during the intervention in comparison to baseline. Based on the scoring system described in the Dietary data analysis section and Table 1, where 100 would indicate ideal dietary adherence, the Total Dietary Adherence score significantly increased (*p* < 0.001) for participants during the Huel intervention to 80.1 ± 23.8 vs. pre-intervention 63.4 ± 15.9.

**FIGURE 11:**
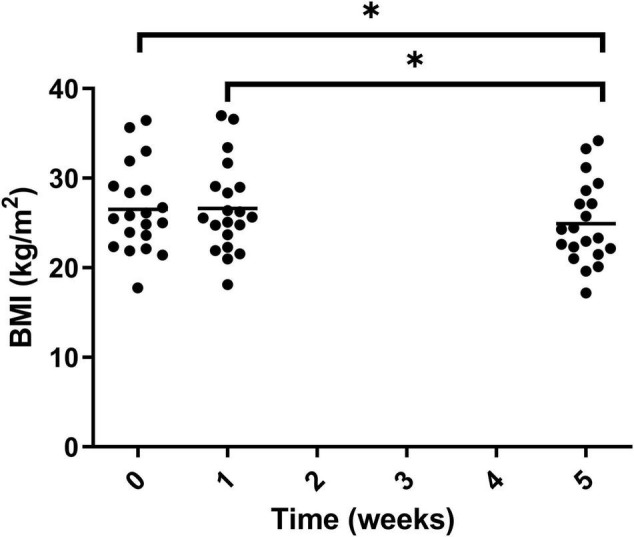
BMI of participants at the three timepoint measured during the trial. Week 1 represents a second measurement after a control week of normal diet. Week 5 represents a third measurement after 4 weeks of a 100% Huel diet. There was a significant difference between weeks 0 vs. 5 and 1 vs. 5. Asterisks denote statistical differences between means.

The BMI of participants was calculated at each of the visits and significantly decreased after the intervention period, –1.6 ± 0.9 from week 0 to week 5. The statistical decrease was seen from both week 0 to week 5 and week 1 to week 5 (Figure 11). This was equivalent to 6.0% ± 3.4 reduction in BMI, taking the mean of the study population from 26.51 ± 4.88 to 24.91 ± 4.62 kg/m^2^.

**FIGURE 12:**
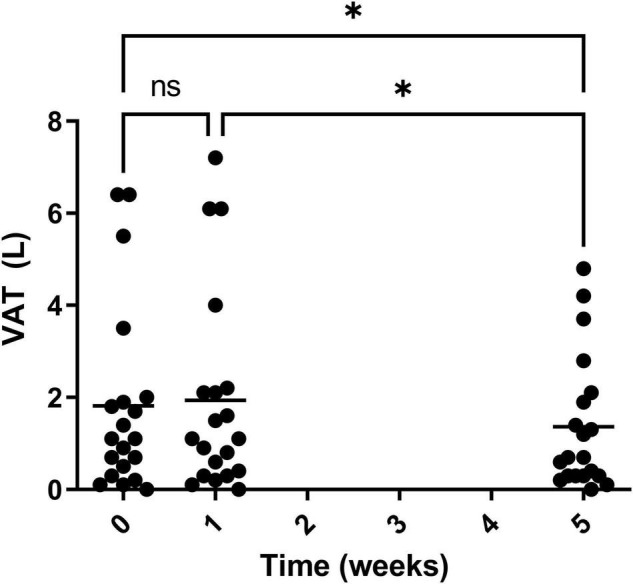
Visceral adipose tissue (VAT) of participants throughout the trial. Week 1 represents a second measurement after a control week of normal diet. Week 5 represents a third measurement after 4 weeks of a 100% Huel diet. The mean VAT of all the participants who completed the trial, with asterisks denoting statistical differences.

Visceral adipose tissue was also calculated during the study and a significant decrease was observed for both week 0 vs. 5 and weeks 1 vs. 5 (Figure 12). Over the course of the trial, three of the 20 participants showed an increase in VAT after 4 weeks on Huel, as compared to Week 0. Thirteen participants showed a decrease in VAT after 4 weeks on Huel, as compared to Week 0, with four participants showing no change. The mean change in VAT for participants was –0.45L ± 0.76.

**FIGURE 13:**
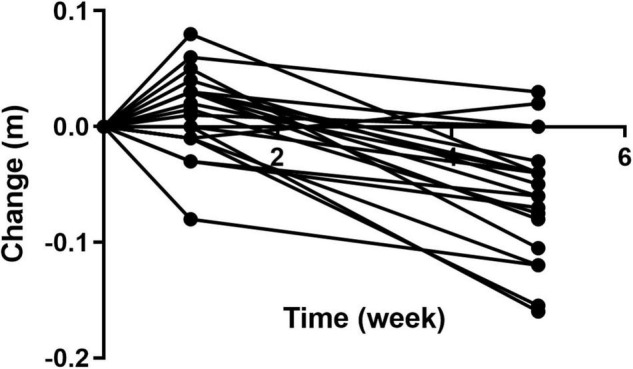
Change in waist circumference of participants throughout the trial as compared to Week 0. Week 1 represents a second measurement after a control week of normal diet. Week 5 represents a third measurement after 4 weeks of a 100% Huel diet.

Over the course of the trial, two participants of 20 showed an increase in waist circumference (m) after four weeks on Huel, as compared to Week 0 (Figure 13). Sixteen participants showed a decrease in waist circumference (m) after four weeks on Huel, as compared to Week 0, with two participants showing no change compared to week 0. The mean change in waist circumference for participants was –0.060m ± 0.053.

## Discussion

The 22 micronutrients measured in this study were compared to their two baseline measurements, with each participant acting as their own control. Of the 22 analytes measured, eleven statistically changed after the intervention period with five significantly increasing and six significantly decreasing.

The current before and after, preliminary intervention study highlighted that elective intake of Huel over a four-week period resulted in changes to nutritional status, health parameters and energy and nutrient intake. The suggested intake of Huel during the study was based on their own baseline week energy intake. The amount of Huel required to meet this was recommended to the participants. While dietary data suggest that all participants complied to only consuming Huel during the intervention period, data also suggest that their total intake was consistently below recommended energy intake. The energy restriction over a 4-week period may have resulted in improvement in some parameters of health (particularly body weight and cardiometabolic parameters), as has been previously noted in other energy restriction-based interventions (14, 15), albeit with longer intervention periods. However, restricted energy intake would also have the potential to limit adequate consumption of other nutrients. Dietary intake at baseline did not meet energy or nutrient recommendations for most participants. The intervention improved total adherence scores and adherence to most nutrient recommendations, which aligned with blood biomarker improvements noted post intervention, despite energy intake appearing to be consistently low.

Total cholesterol and non-HDL cholesterol were parameters that significantly decreased after the intervention. Huel is a plant based product and therefore contains a low amount of saturated fat (5% of energy intake) but will contain phytosterols from ingredients such as flaxseed which have been shown to reduce LDL cholesterol (16, 17). The changes in LDL cholesterol level noted appear to be commensurate with the observed change in total and non-HDL cholesterol. These parameters are widely accepted to represent markers of cardiovascular health (4). HbA1c concentrations were also improved, suggesting an improvement in glycaemic control for participants. The Huel formulation is plant based and contains relatively low amounts of saturated fat when compared to on Western diets which on average has 8.4% energy intake from saturated fats but can contain over 16% energy intake from saturated fats (18). The standardisation of the product’s lipid profile may have supported participants in improving their overall fatty acid intake, while the more-slowly digested, higher quality carbohydrate content may have had resulted in blunting of postprandial glycaemic responses expected to benefit blood glucose control (19, 20). Previous studies have suggested that switching to an *ad libitum* whole food plant based diet over 8-weeks to one year resulted in significant improvements to blood lipid profiles and glycaemic control (21, 22). While similar effects may have been expected within this study, the authors believe that the restriction of energy intake noted here is the most likely reason for improvements in the health markers observed. The inclusion of acerola cherry powder in Huel may also have a antihyperglycemic effect, as extracts from acerola cherry powder have been shown to reduce α-glucosidase activity, limiting the uptake of glucose from carbohydrate digestion (23).

Initially 58% (11/19) of participants had sub optimal vitamin D status during the first two measurements of the study. The percentage of participants who were below the recommended level of vitamin D decreased after the intervention period. However, the study was conducted from February 2021 until July 2021 so the increase in exposure to sunlight may have the potential to increase the levels of vitamin D. Although the majority of participants completed the study by April 2021 where increase in day length might not have sufficient effect on vitamin D levels in participants. The levels of vitamin D in Huel are 80% of nutrient reference value (NRV) in each 100g. This would be the equivalent of one portion of Huel (two standard scoops), with all participants regularly consuming more than this, they would achieve their recommended daily intake consistently over the course of the intervention.

Data from dietary records would suggest that all participants carefully followed the Huel intervention, with all records noting that individuals only consumed Huel and acaloric beverages during the study. The improvements in iron intake and haemoglobin levels post intervention therefore almost certain to be a result due to the Huel formulation, with 57% of nutrient reference value being present in 100 g of powder. Therefore, two servings of Huel per day would be sufficient to meet recommended dietary intake. No supplementation of Huel Power was listed as an ingredient as iron would be naturally present in the plant-based ingredients, such as oats flaxseed and tapioca flour. Measurable improvements in haemoglobin status were noted despite all iron in Huel being as non-haem sources from the whole plant ingredients and the potential that other dietary factors (e.g., phytic acid) within the formulation has the potential to reduce iron absorption (24).

Similarly, markers of iron status improved within the four-week trial in parallel with higher dietary intake of iron among participants in comparison to their baseline dietary habit. While relatively quick changes [e.g., less than a week (25)] have been suggested for iron status markers in supplementation studies targeting individuals with, the current cross-section of participants all started the study with normal iron status. While relatively small in overall effect (in this intervention period), the increased in haemoglobin and blood iron concentrations could be important in ensuring individuals who are susceptible to sudden loss of iron (e.g., as a result of blood loss, menstruation or other increase in bodily iron turnover) that might move them toward optimal iron status. It is also positive that this minor improvement in iron status was seen despite the potential challenges with iron bioavailability in plant-based foods in part due to the presence of phytic acid (24). However, the high levels of vitamin C are known to enhance iron absorption as well as other minerals (26, 27). Levels of calcium are relatively high in Huel powder at 75% dietary reference value per 100 g serving in Huel powder negating the bioavailability issues due to the presence of phytic acid.

Levels of vitamin A and E both statistically decreased over the course of the study. For both the vitamins there were participants who started the study with higher than recommended levels of the vitamins, 1 of 19 for vitamin A and 2 of 19 for vitamin E, but after the intervention all of the participants were within the desired range. Huel Powder contains 23 and 25% for vitamin A and E respectively, of the nutrient reference value per 100 g serving. Potentially not all participants would have consumed four or more portions of Huel per day so could potentially have lower than recommended intake.

Cellular uptake of vitamin E potentially shares similar mechanisms to that of cholesterol, and with lower amounts of cholesterol present in Huel, the uptake of vitamin E may be at its optimum as there is less competition (28) and with participants refraining from drinking alcohol for the duration of the intervention, and ethanol intake having a negative effect on vitamin A uptake (29), then maintenance of the appropriate levels of vitamins A and E may have been achievable.

In each 100 g serving of Huel, 35 and 36% of dietary reference value intake of potassium and selenium respectively were present. All participants started the study and completed the intervention within the recommended range of potassium, but the mean level did statistically decrease after the intervention compared to the two baseline measurements. For selenium, however, the average statistically increased post intervention compared to the two baseline measurements. There was one participant below the recommended level before the intervention, but none were post intervention. Therefore, if participants were consuming lower then NRV amounts of potassium due to lower Huel intake then it would be expected that there would also be lower selenium intake as the percentage NRV are similar. Therefore, the bioavailability of potassium may be different *in vivo* to the predicted NRV. Recommended intake values have been generated from data using supplementation rather than foods, with limited studies looking into the bioavailability of potassium from food sources (30). Potassium from potatoes has been shown to be like that of potassium gluconate supplements but with limited data in other food sources (31).

Common sources of vitamin B12 are often of animal nature and deficiencies in vitamin B12 are seen in consumers of low animal source foods (32). Huel Powder contains ingredients of only plant origin but still contains 32% of nutrient reference value per 100 g. The absorption of vitamin B12 is complex and involves the complexation with multiple factors throughout the gastrointestinal tract before absorption in the terminal ileum (33). The potential for prolonged retention in the stomach due to the viscosity (34) of Huel and the retention in the small intestine due to nutrient density (35) may prolong the time required for the complexation and degradation stages to complete, optimising the bioavailability of the vitamin. One participant began the study with lower than recommended levels of circulating vitamin B12 but after the intervention all participants were above this minimum recommended level indicating that even if all participants did not achieve their NRV intake, they could maintain their vitamin B12 status.

### Strengths, Limitations, and Future Work

These pilot findings were intended to inform future larger, longer studies on the impact of Huel on health outcomes. While the primary outcomes of the current study were changes to nutritional status markers after intervention, it appears that the most striking impacts in outcomes measures were on body weight, lipid status and glycaemic control. While these findings suggest a tendency that the Huel intervention resulted in an improvement in broad measures of cardiometabolic health, it must be noted that participants were not specifically recruited from an at-risk population in terms of current body weight status, cardiovascular health, or glycaemic control. Baseline values suggest that most participants were generally healthy, with only 37% (7/19) having high total cholesterol and 63% (12/19) falling into the excess body weight category by BMI. However, if similar reductions in elective energy intake and improvements in overall nutrient intake were seen in at-risk populations, similar outcomes might be expected. Further interventional studies are required in more targeted populations (including pre-screening, appropriate sample sizes and control groups or crossover design) to help support the preliminary findings noted in the current study.

The findings of elective energy restriction during the Huel intervention period appear to be particularly pertinent in designing future studies. It is possible that participants signed up to this trial with the idea of reducing their body weight/improving their health, as has been suggested to be a common motivator for participants on human nutrition studies (36). Ensuring energy matching of interventions based on Huel or other similar products is important to be able to differentiate between impacts on health outcomes affected by energy restriction versus those that might be an impact of a more positive overall compositional profile than habitual dietary intake. While our initial findings suggest Huel intake tended to improve estimates of dietary adequacy and nutritional status, it is also important to explore whether issues with dietary diversity (in terms of sensory aspects of the products) could also influence individuals to reduce overall food and energy intake, as has been suggested in other studies based on liquid meals (37).

The Huel range is not intended to be the sole source of food for users. Therefore, there is also clearly a need for additional studies based on different levels of Huel integration into a standard diet, particularly to explore whether different “dosages” could improve overall dietary habit and health outcomes. Due to the time length in order to see an impact on health outcomes, it is unlikely that such a study could be carried out in a live-in cohort (38). Provision of all other meals (along with Huel products) to be consumed during the intervention period provides a way of controlling or energy-matching dietary intake of free-living individuals under different treatment arms but would present a series of logistical issues (39). Less management of the non-Huel components of dietary intake in future studies would require careful evaluation of dietary intake (particularly in comparator timepoints or arms) but also would seem more likely to align with the “real world” impact of using a product like Huel within a wider dietary habit.

## Conclusion

These preliminary findings suggest that an intervention solely based on intake of Huel tended to improve overall dietary intake in this cohort of participants, with further improvements to markers of cardiometabolic health and nutritional status also noted. The voluntarily elected lower energy intake for participants was seen through the four-week intervention when compared to baseline measurements is likely to have driven improvements in BMI, waist circumference and visceral adipose tissue. Being able to lose weight and maintain adherence to dietary requirements could be useful in the clinical setting and warrants further investigation.

## Data Availability Statement

The raw data supporting the conclusions of this article will be made available by the authors, without undue reservation.

## Ethics Statement

The studies involving human participants were reviewed and approved by Newcastle University Faculty of Medical Science Ethical Committee. The patients/participants provided their written informed consent to participate in this study.

## Author Contributions

MW, PC, JP, and RW helped formulate the research question and design the study. MW, KS, and PC carried out the study. MW, PC, IB, and JP analysed the data and interpreted the findings. MW, PC, IB, JP, and RW wrote the manuscript. All authors read and approved the final manuscript.

## Conflict of Interest

This study received funding from Huel Ltd. The funder had the following involvement with the study: design of research question and input in the design of the study. MW, PC, IB, and JP are co-founds and shareholders in Aelius Biotech. The remaining authors declare that the research was conducted in the absence of any commercial or financial relationships that could be construed as a potential conflict of interest.

## Publisher’s Note

All claims expressed in this article are solely those of the authors and do not necessarily represent those of their affiliated organizations, or those of the publisher, the editors and the reviewers. Any product that may be evaluated in this article, or claim that may be made by its manufacturer, is not guaranteed or endorsed by the publisher.
